# A fast and responsive turn-on fluorescent probe based on a quinone conjugated alkoxy derivative for biothiols and a cellular imaging study

**DOI:** 10.55730/1300-0527.3702

**Published:** 2024-11-11

**Authors:** Ulviyye NEMETOVA, Ayşe Nur ÖNEM, Alev ER, Sefa ÇELİK, Ayşen E. ÖZEL, Sevim AKYÜZ, Mustafa ÖZYÜREK, Sibel ŞAHİNLER AYLA

**Affiliations:** 1 Division of Organic Chemistry, Department of Chemistry, Faculty of Engineering, İstanbul University-Cerrahpaşa, İstanbul, Turkiye; 2 Division of Analytical Chemistry, Department of Chemistry, Faculty of Engineering, İstanbul University-Cerrahpaşa, İstanbul, Turkiye; 3 Department of Physics, Faculty of Science, İstanbul University, İstanbul, Turkiye; 4 Department of Physics, Faculty of Science and Letters, İstanbul Kültür University, İstanbul, Turkiye

**Keywords:** Biothiols, 2, 3-dichloro-5, 8-dihydroxynaphthalene-1, 4-dione, fluorogenic probe, bioimaging, molecular modeling, molecular docking

## Abstract

The detection of intracellular biothiols (cysteine, N-acetyl cysteine, and glutathione) with high selectivity and sensitivity is important to reveal biological functions. In this study, a 2-(2-methoxy-4-methylphenoxy)-3-chloro-5,8-dihydroxynaphthalene-1,4-dione (DDN-O) compound **(3)** was newly synthesized and used as a fluorogenic probe (detector molecule) in the fluorometric method for the rapid, highly selective, and sensitive determination of biothiols. The intensity values (λ_ex_ = 260 nm, λ_em_ = 620 nm) of the product were measured by adding biothiols to the reaction medium at varying concentrations and the glutathione equivalent thiol content values of each biothiol were calculated. Using compound 3, glutathione as the reference biothiol was detected in the linear concentration range of 10–70 μM and the LOD value was found to be 0.11 μM. Biothiol detection with structurally simple compound 3 was performed at the cellular level within 1 min and the probe was also successfully used in bioimaging with low cytotoxicity. It was concluded that this probe can serve as an alternative to existing fluorescence-based biothiol probes with applications in rapid biothiol detection at the cellular level for biological functions. To evaluate the molecular structure of 3, conformational analysis was performed using the PM3 semiempirical method. The most stable obtained molecular geometry was then optimized at the DFT/wb97xd/6-311++G(d,p) level of theory. Frontier molecular orbitals (HOMO and LUMO) and molecular electrostatic potential map analyses were performed for the optimized structure. Molecular docking studies demonstrated the interactions of 3 with HAS (1AO6) and FhGST (2FHE) target proteins.

## 1. Introduction

Sulfur-containing amino acids such as glutathione (GSH), N-acetyl cysteine (NAC), and cysteine (CYS) are nonprotein biothiols, constituting important compounds that play vital roles in many different pathological activities in living organisms [1]. Changes in the concentration levels of these biothiols in living cells are associated with symptoms that can cause disruptions in cells and even in organs. GSH is a biothiol that plays a role in intracellular redox homeostasis and acts as a powerful antioxidant against reactive oxygen species (ROS), and changes in GSH levels are closely related to diseases such as cancer and Parkinson’s [2]. On the other hand, a decrease in CYS level may cause disorders such as slow growth [3]. Moreover, there are certain correlations between total thiol content (TTC) and complications from hypertension, chronic renal failure, and type 2 diabetes mellitus observed in preeclampsia. Therefore, monitoring changes in the concentration levels of biothiols in cells can provide important data for the early diagnosis and treatment of TTC-related diseases.

In the literature, fluorescent probes with a wide range of fluorophore groups have been introduced for use in cellular biothiol detection, offering a number of advantages including high sensitivity and selectivity, practicality, visualization, and noninvasiveness [4]. The fluorometric determination of biothiols is carried out using various signal transduction processes such as photoinduced electron transfer (PET), Förster resonance energy transfer, or intramolecular charge transfer accompanying the reaction mechanism (e.g., nucleophilic substitution, thiolysis, nucleophilic addition, cyclization, or Michael addition) between fluorophore groups in the probe structure and analyte (i.e. the biothiol) [5]. One of the most commonly used mechanisms in fluorescence-based determination methods for fluorogenic probe design is nucleophilic substitution [6,7]. On the other hand, these probes present difficulties in terms of the rapid detection of changes in biothiol concentration at the cellular level and the dynamic monitoring of such changes [8].

Quinone group compounds are present in many natural products and can also be synthesized synthetically [9–11]. The structural modification sites of quinones have commonly been based on *N*-, *S*-, or *O*-substitutions [12–14]. In addition to these features of quinones, they are also used as reaction sites because of their characteristic of being excellent Michael acceptors [15]. The mechanism for that reaction involves the Michael addition of a nucleophile to the reacting quinone compound under an oxygen atmosphere [16,17]. Although the synthesis and application of quinone-based sensitive probes have been addressed in the literature, studies on these topics are still relatively limited. In the present study, we synthesized an effective fluorescent probe, 2-(2-methoxy-4-methylphenoxy)-3-chloro-5,8-dihydroxynaphthalene-1,4-dione (DDN-O) (3), obtained from 2,3-dichloro-5,8-dihydroxynaphthalene-1,4-dione (1) and 2-methoxy-4-methylphenol (2). Spectroscopic techniques including ^1^H NMR, ^13^C NMR, FTIR, UV-Vis, and mass spectrometry were used to identify the chemical structure of compound 3. Compound 3 itself does not have any fluorescence properties, but when biothiols were introduced into the system, the fluorescence intensity increased due to the formation of the DDN-O-biothiol product (DDN-OG) by nucleophilic substitution. In this context, an off–on fluorescence strategy was designed for the highly sensitive and selective determination of biothiols. Furthermore, the in vitro applicability of compound 3 was evaluated via fluorescence imaging of GSH performed using the A549 human lung cancer cell line with low cytotoxicity.

## 2. Materials and methods

All reagents were of analytical reagent grade unless otherwise stated. The solutions used in the synthesis and method optimization stage, the preparation procedures applied for these solutions, and the related instruments and spectra are described in the Supplementary Information.

### 2.1. Synthesis of the probe (3)

To a solution of 2,3-dichloro-5,8-dihydroxynaphthalene-1,4-dione (1) (0.5 g, 2.0 mmol) in 20 mL of DMSO, 2-methoxy-4-methylphenol (2) (0.27 g, 2 mmol) was added. Na_2_CO_3_ was subsequently added and the solution was stirred vigorously. The resulting product was obtained after approximately 35–40 min and the suspension was stirred for a further 5–6 h. After a red solid formed, the reaction mixture was extracted with chloroform (60 mL). The organic layer was dried over anhydrous sodium sulfate and evaporated in vacuo. The crude product was purified by column chromatography on silica gel with the CHCl_3_ mobile phase to give a red solid (0.60 g, 83%).

Dark red solid; mp: 140–142 °C. Yield: 0.59 g (83%). R*f*: 0.60 [CHCl_3_]. IR (ν, cm^−1^): 3265 (OH), 2973, 2925, 2871, 2830 (−CH), 1664 (C=O), 1572 (C=C). ^1^H NMR (499.74 MHz, CDCl_3_, ppm): δ 12.77 and 12.13 (s, 1H, −OH), 2.25 (s, 3H, −CH_3_), 3.65 (s, 3H, −OCH_3_), 6.25–7.18 (m, 8H, H_arom_). ^13^C NMR (125.66 MHz, CDCl_3_, ppm): δ 20.39 (−CH_3_), 55.35 (−OCH_3_), 158.04, 157.39, 154.40, 147.69, 142.56, 134.23, 129.44, 128.76, 128.64, 127.83, 120.59, 120.56, 117.12, 112.88, 109.48 (C_arom_, CH_arom_), 179.13, 180.61 (C=O). UV-Vis (C_2_H_5_OH) λ (logɛ) = 545 (3.39), 508 (3.63), 345 (2.20), 284 (3.81) nm. UV-Vis (CHCl_3_) λ (logɛ) = 545 (3.28), 345 (2.10), 284 (3.71) nm. MS [+ESI]: m/z = 357 [M-2H]^−^. Anal. calc. for C_18_H_13_ClO_3_ (360.65): C, 59.93%; H, 3.63%. Found: C, 59.88%; H, 3.78%.

### 2.2. Fluorescence detection of biothiols

The probe (0.04 mL, 10 mM), EtOH (1.96 mL), 0.1 M HEPES ((2 – x) mL, pH 7), and biothiol solution (x mL) were added in that order to obtain a mixture with final volume of 4.0 mL (T = 25 °C). Intensity at 620 nm was recorded after 1 min of mixing samples with reagents. The calibration curves (intensity vs. concentration plots) of each biothiol were constructed under the described conditions. Synthetic mixtures of the biothiols were prepared at suitable volume ratios and those mixtures were subjected to analysis. The theoretical GSH-equivalent TTC (GETC) value of the synthetic mixture solution (expressed as μM GSH) was calculated by multiplying the GETC coefficient of each biothiol constituting the mixture with the final concentration (in μM GSH units) and then summing the products.


(1) $${\text{TTC}}_{\text{expected}}=({\text{GETC}}_{1}\times {\text{conc.}}_{1})+({\text{GETC}}_{2}\times {\text{conc.}}_{2})+\ldots ({\text{GETC}}_{\text{n}}\times {\text{conc.}}_{\text{n}})$$

(2) $${\text{TTC}}_{\text{experimental}}=\frac{\text{Intensity }(\text{total})\pm \text{intercept}}{{\text{slope}}_{\text{GSH}}}\times 10^{3}$$

### 2.3. Cytotoxicity assay

#### 2.3.1. Cell culture

The A549 human lung cancer cell line was cultured under standardized cell culture conditions (5% CO_2_, 95% humidity, 37 °C) in 75-cm^2^ flasks. The growth medium was Dulbecco’s modified Eagle medium supplemented with 10,000 U/mL penicillin, 10,000 U/mL streptomycin, 10% (v/v) fetal bovine serum (FBS), and L-glutamine, all obtained from Lonza (Basel, Switzerland). Cells were passaged biweekly.

#### 2.3.2. Cell viability assessment via MTT assay

The cytotoxic effects of the studied compounds against both cancerous and noncancerous cell lines was investigated using the MTT assay [18]. MTT (3-[4,5-dimethylthiazol-2-yl]-2,5-diphenyl tetrazolium bromide) is a soluble tetrazolium salt, which is converted to an insoluble purple formazan by the succinate dehydrogenase enzyme present in the mitochondria of living cells. In the present study, 8000 cells were seeded in 96-well plates and incubated for 48 h under standard incubator conditions. After incubation, the medium was substituted with treatment medium and the cells were exposed to various experimental and control compounds. Following the incubation period, the medium was removed and each well was treated with 110 μL of MTT solution (10%, 5.0 mg/mL PBS). After 4 h of incubation, the formazan crystals were dissolved using 100 μL of SDS solution (1.0 g of SDS in 10 mL of PBS, 0.01 M HCl), and the plates were left in the incubator for 24 h. Results were determined by measuring the absorbance at 570 nm. The data were compared to those obtained from the control group.

### 2.4. Confocal fluorescence imaging of compound 3

The A549 cell line was utilized in a comprehensive experiment involving three rounds of seeding for each sample on Chamber 8 slides followed by a 2-day incubation period. Following this incubation, the control group’s culture media were collected while the experimental group’s media, containing samples treated with the probe (10 μg/mL), underwent an additional 2-h incubation after being carefully added to the wells. Concurrently, a subset of tests were conducted in a prior treatment phase entailing the exposure of the cells to GSH (0.5 mM) for a duration of 2 h at 37 °C. Subsequently, the treated cells were subjected to a 2-h incubation period with a probe solution of 10 μg/mL. The cellular constructs were then subjected to two washes with phosphate-buffered saline (PBS). Cell constructs were evaluated via comparative analysis of the outcomes against a results classification system. This evaluation process was facilitated by visualization with the employment of a fluorescent microscope equipped with a red filter [19].

### 2.5. Computational details

The conformational analysis of compound 3 was conducted utilizing computational methods. Conformation analysis was first performed with the PM3 semiempirical method using Spartan06 software [20]. Subsequently, the lowest energy conformer was optimized utilizing Gaussian 16 software [21], employing the DFT/wb97xd/6-311++G(d,p) level of theory [22], and the vibrational wavenumbers of the optimized structure were calculated using the same theory level. Since no imaginary frequency was obtained, the frequency calculation confirmed that the obtained molecular structure was the most stable conformer.

The optimized molecular structure of compound 3 underwent further energy minimization using the YASARA structure program [23] while employing the NOVA force field. Subsequently, energy-minimized 3 served as input for docking simulations. Additionally, receptors sourced from the Protein Data Bank (PDB; IDs 1AO6 and 2FHE) were subjected to energy minimization using the YASARA structure program with the NOVA force field before docking simulations.

Crystallographic data pertaining to human serum albumin (PDB ID: 1AO6) [24] and GST from *Fasciola hepatica* (PDB ID: 2FHE) [25] were obtained from the PDB via the official repository website (http://www.rcsb.org/pdb).

A molecular docking investigation utilized the VINA approach [26] with the YASARA program (v22.9.24). The target protein retrieved was prepared for docking by extracting the ligands and water molecules from PDB files and incorporating polar hydrogen atoms. Kollman charges were assigned to the target. Default parameters were maintained for other settings. Docking simulations were conducted employing a semiflexible docking protocol, maintaining flexibility in the ligand while keeping the target protein rigid.

## 3. Results

### 3.1. Spectroscopic response of fluorogenic probe 3

The synthesis of the new alkoxy substituted compound (3) was focused on the replacement of one chlorine atom in the quinone pharmacophore. The preparation of 3 is illustrated in the Supplementary Information. 2-(2-Methoxy-4-methylphenoxy)-3-chloro-5,8-dihydroxynaphthalene-1,4-dione (3) was synthesized from the reaction of 2,3-dichloro-5,8-dihydroxynaphthalene-1,4-dione (1) and 2-methoxy-4-methylphenol (**2**) in the presence of the Na_2_CO_3_ solution of dimethyl sulfoxide.

The hydrogen signals of the (−OH) protons of compound 3 were assigned to 12.13 and 12.77 ppm as singlets (Figure S1). In addition, the ^13^C NMR spectra of compound 3 displayed a characteristic signal of two carbonyl groups (C=O) at a chemical shift in the range of 180.61 to 179.13 ppm (Figure S2).

The replacement of the alkoxy group was further confirmed by electrospray ionization (ESI) in mass spectrometry measurements; the mass spectra of product 3 exhibited a signal at m/z 358 corresponding to molecule [M-2H]^−^ (Figure S3). The FT-IR spectra of compound 3 revealed the stretching band of the C=O group at 1655 cm^−1^ (Figure S4). The UV-Vis absorption characteristics of the compound in the presence of chloroform or ethanol without GSH were also recorded and *O-*substituted derivatives showed four meaningful absorption bands. Figure S5 presents a sample of the UV spectra for three main different absorption values of 3 in ethanol and chloroform with high-energy bands at approximately 204–2190 nm, intense bands at approximately 262–284 nm, and low-intensity visible bands at approximately 508 and 545 nm. The strong absorption peak centered at 20–220 corresponded to the π–π* transition of the quinonoid ring, while the weak absorption peak centered at 508–594 nm could be attributed to the n–π* transition. These third lower-energy transitions appeared in the visible region of ~500 nm and they could be assigned as alkoxy substituted quinones. The large molar absorption values in this region also supported this conclusion. With decreasing solvent polarity, both peaks with λ_max_ were slightly affected.

### 3.2. Possible sensing mechanism of compound 3

**Figure 1:**
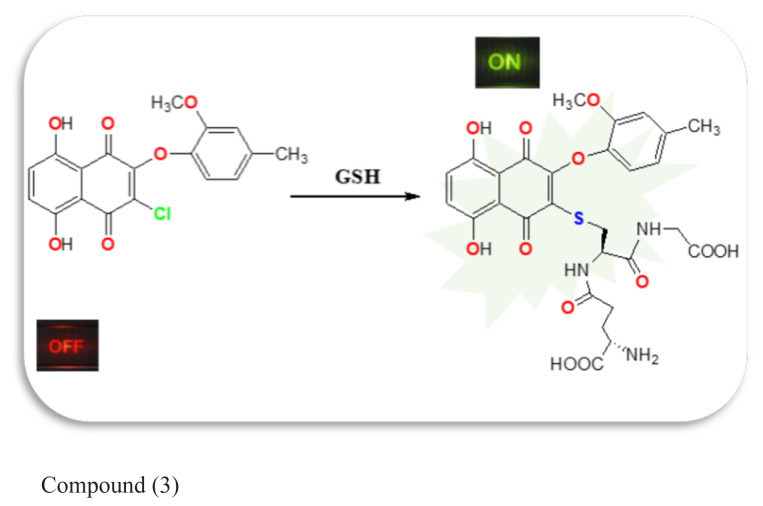
Possible sensing mechanism of the reaction between compound 3 and GSH.

An obvious change in the fluorescence of the probe was observed when biothiol was added. This can be explained by the replacement of the single chlorine atom in compound 3 with the sulfur atom of the biothiol, which has nucleophilic properties, and the formation of the probe. Michael addition occurred between compound 3 and GSH forming a single bond between the S and C atoms. Finally, the glutathionylation product had florescence. Before that, due to the strong electron-withdrawing property of the quinone group, the fluorescence was likely to be quenched by the PET process. An addition to the sulfhydryl group blocked the PET and caused the fluorescence (Figure 1).

### 3.3. Analytical Figures of merits

The basis of the fluorometric method using compound 3, which has high selectivity and sensitivity and responds very fast to biothiols, involves the formation of a fluorescence adduct after the interaction of the probe with biothiols.

**Figure 2:**
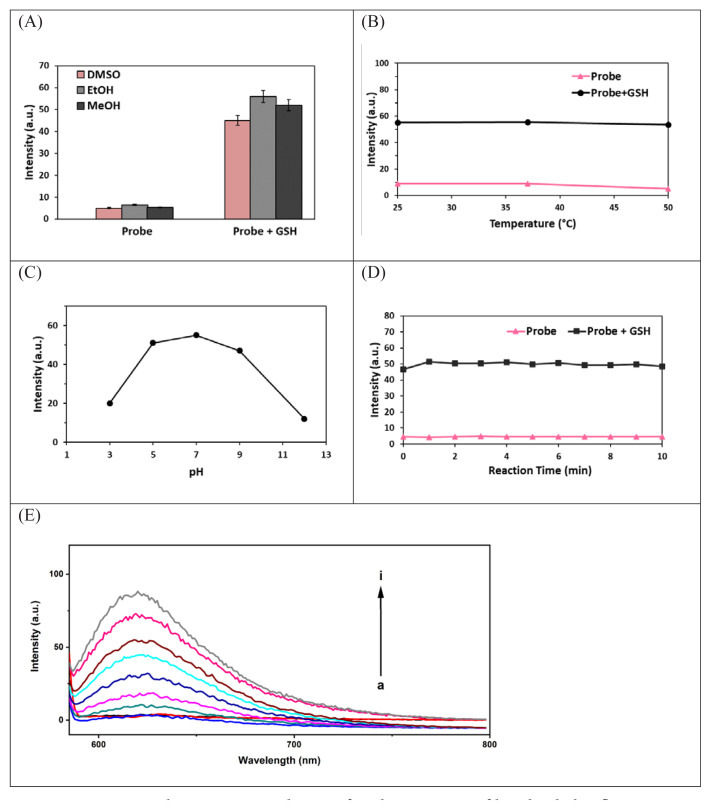
Optimal reaction conditions for the sensing of biothiols by fluorogenic compound 3: effects of the solvent (A), pH (B), and temperature (C) on GSH sensing of probe; reaction kinetics of 3 with/without GSH (D); emission spectrum of **3** (100 μM) versus increasing concentrations of GSH (10–70 μM) (E).

A stable sulfur-substituted product consisting of GSH (50 μM) and compound 3 (0.1 mM) was obtained in the presence of EtOH and achieved maximum fluorescence intensity (Figure 2A). On the other hand, at 25 °C, a plateau was reached for both compound 3 alone and compound 3 + GSH, and the intensity change remained constant (Figure 2B). The effect of pH on the probe in the presence of GSH was studied in the range of 3.0–12.0 under the same experimental conditions used for the proposed fluorometric method. The highest probe intensity was observed at pH 7.0. As displayed in Figure 2C, there was essentially no change in the fluorescence intensity of compound 3 in the absence of GSH within the studied pH range, suggesting that the probe had good stability in a broad pH range. According to the time-dependent fluorescence intensity changes (Figure 2D), the reaction between the probe and GSH was fast, and in the presence of 100 μM GSH, the reaction was completed within 1 min (i.e. an intensity plateau was reached).

Using the relationship of the excellent linearity between the concentrations of GSH in the range of 10 to 70 μM and intensity (Figure 2E), the linear equation for GSH was taken as Intensity (I) = 1.30 × 10^6^c + 0.36 (Figure 2). The slope value of the calibration line gives the molar extinction coefficient of the GSH compound (1.30 × 10^6^ L mol^−1^ cm^−1^). The limit of detection (LOD) and limit of quantitation (LOQ) values of the GSH compound obtained by the fluorometric method were 0.11 and 0.36 μM, respectively. The fluorescence quantum yield (Φ) of the resulting adduct (probe + GSH) was found to be 0.32 with rhodamine 6G (Φ = 0.95 in EtOH) as a reference.

The relative standard deviation (RSD, %), which represents repeatability in the concentration range at which the calibration line is obtained, was calculated as 1.21%. With the developed method, calibration equations involving molar concentration and the intensities of biothiols (GSH, NAC, and CYS) were applied. Table 1 summarizes the linear equations of the standard biothiols (A = mc + n), correlation coefficients (r), linear concentration ranges, and GETC values. The GETC values found for NAC (0.86) and CYS (1.27) were consistent with the findings obtained with the original Elman method (GETC_CYS_: 1.06; GETC_NAC_: 0.93) and the DTNB-Au-NP method (GETC_CYS_: 1.02; GETC_NAC_: 0.98) [27,28]. Furthermore, the LOD values calculated according to the proposed method were lower than the LOD values obtained according to the DTNB-Au-NP and original Ellman methods. In a similar study involving the thiol–halogen nucleophilic substitution mechanism, a low LOD value was obtained with the monochlorinated BODIPY ratiometric fluorescent sensor for GSH detection [29], but the probe that we have developed responds very rapidly to biothiols. Another probe with a similar mechanism has a protocol time of 5 min and lower sensitivity than that of the newly developed probe [30]. The new probe has a very low LOD value, especially for NAC, and this probe could be useful in determining the intracellular concentration levels of biothiols and in the analysis of human plasma samples [31,32].

Although the response time of most fluorescence-based biothiol probes is over 1 min, it appears that compound 3 has lower or comparable detection limits than probes with similar protocol times as given in Table 2. Relatively long detection times limit the use of fluorogenic probes in the analysis of biothiols. Compared to other fluorogenic biothiol probes, compound 3 has specific advantages over other probes, including fast response time, high sensitivity, low cost, and simple operation. The stable fluorescence response toward biothiols at a pH value of approximately 7.0 is also favorable for detection experiments. Compound 3 can function in the physiological pH range present within cellular environments. Comparative data for the newly developed probe and some other fluorescent probes are provided in Table 2, indicating that the proposed probe is promising for practical analysis.

Synthetic mixtures consisting of binary combinations of standard biothiols were prepared and their thiol capacities were determined using the developed method (Supplementary Information, Table S). It was observed that the theoretically expected capacity values for synthetic mixtures and the experimentally determined capacity values (μM GSH) were consistent with deviation of only ±6.8%. In light of these results, it can be said that the sum of the individual capacities of the biothiols contained in the biothiol mixture determines the total thiol capacity of the mixture.

As shown in Table 3, in terms of the amounts of GSH added and values experimentally determined for binary mixtures of GSH and compounds expected to exhibit possible interference properties, it was confirmed by recovery (REC) values with relative errors between −1.6 and −4.6 that the measured interferences did not have a significant effect on the fluorescence intensity of the GSH.

By adding standard biothiols to FBS with the developed method, the reproducibility and recovery of the method were investigated. The relative standard deviation (%, RSD) values of intensity measurements in the concentration range in which the trials were performed reflected the reproducibility of the method and the highest value was found to be 4.24% (Table 4). REC values were between 93.9% and 105.1%.

### 3.4. Cytotoxic effects of compound 3

**Figure 3:**
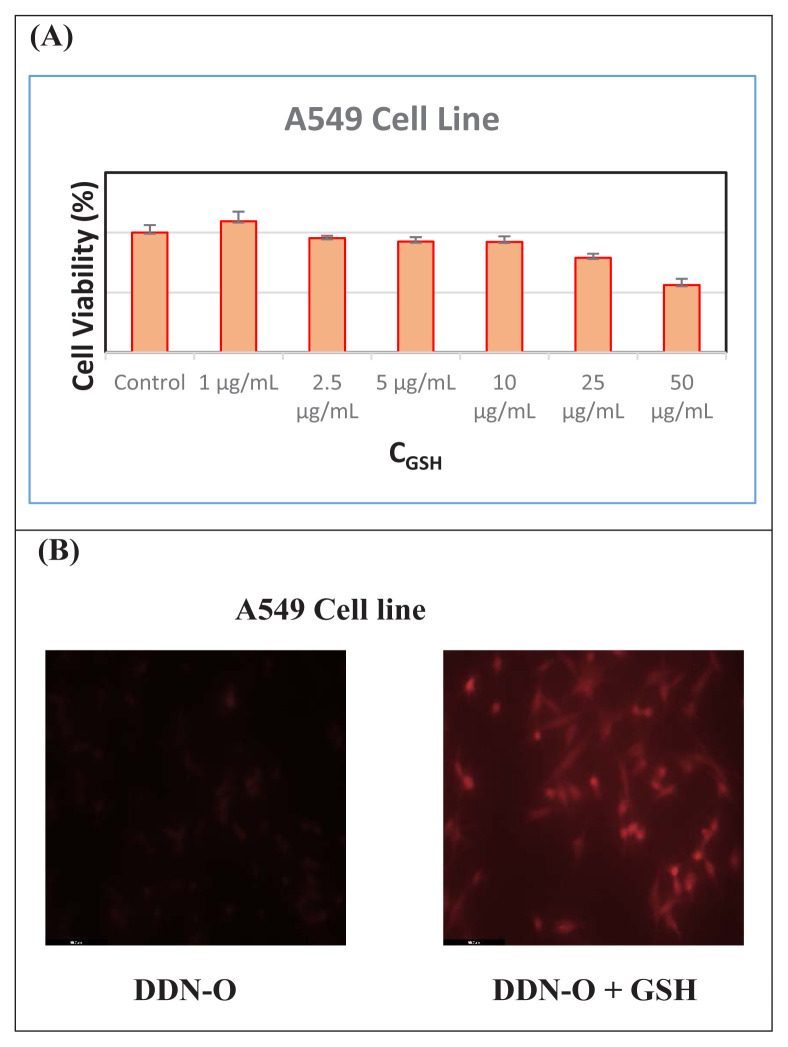
(A) Cytotoxicity of the probe against A549 cells. (B) Fluorescence imaging of A549 cells treated with the probe at 10 μM and of the probe (10 μM) treated with GSH solution.

For the imaging of GSH in living cells, cytotoxic effects in A549 cells were evaluated using the MTT assay in a dose-dependent manner. After 24 h of incubation of the cells with the probe at various concentrations of 1–50 μg/L, cell viability percentages were found to be approximately 82.65%–94.25%, as shown in Figure 3A. These results indicate that the probe has low cytotoxicity against A549 cells with great potential for a variety of biological applications.

### 3.5. Fluorescence imaging of the probe in A549 cells

Based on our findings of low cytotoxicity, we subsequently examined whether the synthesized probe could be applied for the detection of biothiols in cells. As shown in Figure 3B, when A549 cells were treated with the probe at a concentration of 10 μM for 2 h, no detecTable fluorescence was observed. However, when the cells were cultured with the probe in addition to GSH solution, fluorescence was observed.

### 3.6. Molecular electrostatic potential

Molecular electrostatic potential (MEP) characterizes attributes including dipole moment, electronegativity, and partial charges that are intrinsic to a molecule, providing a direct way to evaluate the molecule’s polarity. MEP is a valuable tool for discerning hydrogen bonding interactions by pinpointing electrophilic and nucleophilic regions within the molecular structure.

Positive electrostatic potential regions are areas with reduced electron density, indicative of repulsion toward a positive charge (proton). Conversely, negative electrostatic potential areas, with features like lone pairs and pi-bonds, are regions in which a positive charge (proton) would experience attraction.

The MEP of bioactive compound 3 offers important insights into potential molecular locations that may be engaged in interactions with the target protein’s amino acid residues. Using the DFT approach at the wb97xd/6-311++G(d,p) level of theory, the MEP of the optimized structure of 3 was determined and is presented in Figure S6, where red represents negative regions and blue represents positive regions. The potential difference between −2.035 V (dark red) and 2.035 V (dark blue) is represented by the color code of 3, which ranges from −0.07478 AU (dark red) to 0.07478 AU (dark blue). Patches with positive potential values, which are mostly located on hydrogen atoms and suggest potential nucleophilic attack sites, are seen in Figure S6. Additionally, there are areas of negative potential around the oxygen atoms that may be targets of electrophilic assault.

### 3.7. Frontier molecular orbital analysis

A molecule’s interactions with other species are assessed using the energies of its highest occupied (HOMO) and lowest unoccupied (LUMO) molecular orbitals. Their energy gap allows one to estimate the wavenumbers at which the molecule will emit or reflect, indicating the reactivity of the molecule. At the DFT/wb97xd/6-311++G(d,p) level of theory, the HOMO–LUMO gap was expected to be 6.375 eV. In Figure S7, the frontier orbitals of 3 are schematically diagrammed. The presence of charge transfer interactions inside the molecule is indicated by the low HOMO and LUMO energy gaps.

### 3.8. Molecular docking

Human serum albumin (HSA) is the predominant serum protein, accounting for approximately 60% of total serum protein contents. HSA serves as a carrier for numerous exogenous and endogenous molecules, facilitating their binding and transportation. Structurally, HSA is a single-chain polypeptide consisting of 585 amino acid residues and it adopts a characteristic heart-shaped three-dimensional conformation [24]. Its structure can be subdivided into three alpha-helical subunits, denoted as domains I, II, and III, each comprising two subdomains labeled as A and B [39]. HSA has a complex structure that includes three domains connected by 17 disulfide bridges [40]. Within HSA, Sudlow sites I and II serve as the primary ligand-binding regions, localized in subdomains IIA and IIIA, respectively. Furthermore, an additional ligand-binding site has been identified within subdomain IB [41]. The entrance to site I is characterized by the presence of basic residues including Lys195, Lys199, Arg218, and Arg222, whereas its base displays hydrophobic properties.

Moreover, Trp214, a pivotal residue for structural characterization, is present within site I. Site II, relatively smaller in size, primarily consists of hydrophobic residues and facilitates the binding of hydrophobic pharmaceutical compounds. HSA exhibits an affinity for diverse drugs, including thiazolidinediones [42]. Both endogenous (heme, bilirubin, etc.) and exogenous (phenylbutazone, warfarin, etc.) ligands have been shown to bind to HSA at sites I and II according to their crystal structures [43–46]. Serum protein binding affects the pharmacodynamic and pharmacokinetic characteristics of ligands. Since a drug’s pharmacokinetic characteristics, such as uptake rate and clearance, depend on how it interacts with HSA, it is crucial to consider the binding affinity of proposed drugs [47].

The primary class of detoxifying enzymes derived from parasitic helminths are called glutathione S-transferases, or GSTs. They can be considered for use in the development of chemotherapy and vaccines and *Fasciola hepatica* GSTs serve as a useful vaccine against fasciolosis in sheep and cattle [25]. Various helminth species, including nematodes, digeneans, and cestodes, are known to produce GSTs [48]. GSTs constitute a predominant class of detoxifying enzymes in parasitic helminths and they are potential targets for chemotherapy and vaccine development. Notably, GSTs derived from *Fasciola hepatica* demonstrate efficacy in the vaccination of sheep and cattle against fasciolosis [25]. Since helminthic GSTs are the primary detoxification enzymes in these organisms, there is interest in them as potential targets for immunological and chemotherapeutic interventions [49].

**Figure 4:**
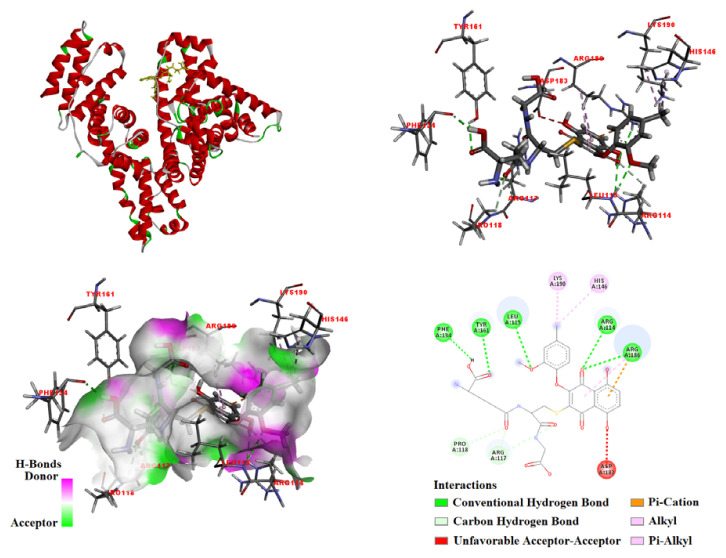
Docking results for compound 3 with human serum albumin (PDB ID: 1AO6). The interacting residues are indicated.

In the present study, the optimized compound (3) was subjected to molecular docking with HSA (PDB ID: 1AO6) to identify its favored binding site and modes (Figure 4). Upon docking, the optimized ligand (3) was observed to bind predominantly within the **3**-binding pocket of HSA, primarily stabilized by the establishment of hydrogen bonds with specific residues. The calculated binding energy for the interaction between 3 and HSA (1AO6) was determined to be −8.448 kcal/mol.

As illustrated in Figure 4, the docked molecule (3) established five conventional hydrogen bonds with the albumin residues at Arg114 (2.7 Å), Leu115 (2.32 Å), Phe134 (2.18 Å), Tyr161 (2.37 Å), and Arg186 (2.98 Å). Additional interactions included a pi–cation interaction with Arg186 (4 Å), unfavorable acceptor–acceptor interaction with Asp183 (2.88 Å), pi–alkyl interactions with His146 (4.61 Å) and Arg186 (4.78 Å, 5.05 Å), alkyl interaction with Lys190 (4.71 Å), and carbon H bond interactions with Arg114 (2.92 Å), Arg117 (2.54 Å, 2.63 Å), and Pro118 (2.93 Å).

**Figure 5:**
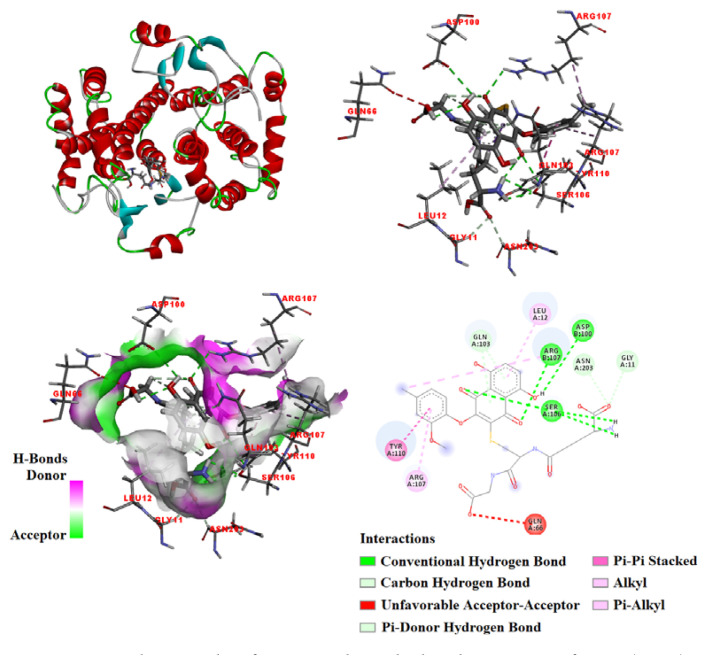
Docking results of compound 3 with glutathione S-transferases (GSTs) from *Fasciola hepatica* (PDB ID: 2FHE), indicating the interacting residues.

Compound 3 forms intermolecular hydrogen bonds with specific residues (Asp100, Ser106, and Arg107) of GSTs derived from *Fasciola hepatica*. As a result, the docked ligand **(3)** forms a stable complex with GSTs from *Fasciola hepatica*. The calculated binding energy for the interaction between 3 and GSTs from *Fasciola hepatica* (PDB ID: 2FHE) was determined to be −7.141 kcal/mol (Figure 5). The interactions with amino acids are as follows: a carbon–hydrogen bond interaction of 2.49 Å in length with Gly11; a pi–alkyl interaction of 5.12 Å with Leu12; an unfavorable acceptor–acceptor interaction of 3 Å with Gln66; a hydrogen bond of 2.94 Å with Asp100; pi–donor hydrogen bond interactions of 2.46 and 3.11 Å with Gln103; hydrogen bonds of 2.22, 2.59, and 2.87 Å with Ser106; a pi–alkyl interaction of 5.25 Å with Arg107(A); a hydrogen bond of 2.64 Å with Arg107(B); an alkyl interaction of 5.1 Å with Arg107(B); a pi–pi stacked interaction of 5.06 Å with Tyr110; and a carbon–hydrogen bond interaction of 2.58 Å with Asn203.

Figure S8 was prepared to compare the optimized structure of 3 to the experimental glutathione ligand conformation retrieved from the PDB file [25].

In this study, we compared the binding modes of glutathione in complex with GSTs from *Fasciola hepatica* (PDB ID: 2FHE) [25] to the binding modes of our compound (3) redocked into the unliganded 2FHE target. Figure S8 shows the comparison of the binding site of compound 3 docked into the unliganded 2FHE target with that of the glutathione ligand in the crystal structure of *Fasciola hepatica* glutathione S-transferase isoform 1 in complex with glutathione (2FHE). The results indicated that the binding site of compound 3 aligned with the experimental findings for the binding site of glutathione in the glutathione S-transferase isoform 1-glutathione complex [25].

## 4. Conclusion

In this study, compound 3 was successfully developed as an ultrafast turn-on fluorogenic probe for the fluorometric determination of biothiols in aqueous and cell samples. The developed compound demonstrated a linear response to standard biothiol compounds including CYS, GSH, and NAC over a wide concentration range. Calibration curves were established for each biothiol and the molar extinction coefficients and linear concentration ranges of these compounds were also determined. The GETC values of these biothiols obtained using the proposed method were compared with the values obtained via the original Ellman method and the DTNB-Au-NP method. General biological sample contents such as amino acids, flavonoids, vitamins, and plasma antioxidants did not interfere with the developed assay method. The proposed method was validated in terms of linearity, totality, reproducibility, and recovery, confirming that the proposed method is reliable and stable. The results for the quantitative detection of total biothiols in different samples and confocal imaging demonstrated the feasibility of the probe for monitoring biothiols in living cells. The in silico screening of 3 against target proteins HSA (PDB ID: 1AO6) and FhGST (PDB ID: 2FHE) revealed high binding affinities (for PDB ID: 1AO6, ΔG = −8.448 kcal/mol; for PDB ID: 2FHE, ΔG = −7.141 kcal/mol), indicating that 3 has good anticancer and anthelminthic properties. We anticipate that our results may offer insights into the bioactivity metabolism of anticancer/anthelmintic drug 3.

## Supplementary Information

### Experimental

The melting point was determined using a Stuart Scientific SPM30 melting point apparatus and is uncorrected. Elemental analyses were performed on a Thermo Finnigan Flash EA 1112 elemental analyzer. IR spectra were recorded with the infra-red (IR) on a JASCO, FT/IR 4700 spectrophotometer in the range of 400 to 4000 cm^−1^. ^1^H-NMR (500 MHz), and ^13^C-NMR (125 MHz) spectra were acquired on a Varian Unity INOVA spectrometer, with CDCl_3_ used as solvent and tetramethylsilane (TMS) as an internal standard. Chemical shifts (δ) were given in parts per million (ppm) and coupling constants were recorded in Hertz (Hz). UV-Vis studies were performed on Perkin Elmer Lambda 35 UV/Vis Spectrometer. The emission spectra and intensity measurements were recorded in a quartz cuvette, using a VARIAN Cary Eclipse spectrofluorometer (Mulgrave, Victoria, Australia).

Mass spectra were obtained on a Thermo Finnigan LCQ Advantage MAX LC/MS/MS spectrometer using the ESI technique. Products were isolated by column chromatography on Sigma silica gel 60, pore size s70–230 μm. Thin-layer chromatography (TLC) was performed on Merck silica gel plates 60 F_254_ and detection was carried out with ultraviolet light (254 nm). All chemicals were reagent grade and used without further purification.

### Chemicals

All reagents were of analytical reagent grade unless otherwise stated. L-glutathione reduced (GSH), N-acetyl-L-cycteine (NAC), DL-cycteine (Cys), L-ascorbic acid (AA), glycine, methanol (MeOH) and ethanol (EtOH were purchased from Sigma-Aldrich (Steinheim, Germany). Sodium hydroxide and albumin (from bovine serum) (BSA) were purchased from Merck (Darmstadt, Germany). HEPES and dimethyl sulfoxide (DMSO) were purchased from Sigma (USA). (+) Catechin (CT) was purchased from Fluka (Buchs, Switzerland). Uric acid (UA) was purchased from Aldrich (Steinheim, Germany).

### Preparation of solutions

The standard solutions at 10 mmol/L concentration of GSH, Cys, NAC, CT, BSA, AA and glycine were all prepared in bidistilled water. The uric acid solution first dissolved with 1 mL 1 M NaOH and then prepared to 10 mmol/L with bidistilled. The stock prob solution (10 mmol/L) was prepared in DMSO. The HEPES buffer at 0.1 mol/L was prepared in bidistilled water. All standard solutions were kept at −20 °C prior to analysis.

Scheme S Preparation of quinone based probe. i: DMSO/Na_2_CO_3._

Figure S1 ^1^H-NMR spectrum of compound (3).

Figure S2 ^13^C-NMR spectrum of compound (3).

Figure S3 Mass spectrum of compound (3).

Figure S4 FT-IR spectrum of compound (3).

Figure S5 Absorption spectra of compound (3) in chloroform (a) and ethanol (b).

**Table S:** Comparison of theoretical and experimentally found total thiol contents (TTC) of synthetic mixtures with respect to the compound (3) (N = 3).

Composition of mixture	TTC _component_ (μM GSH)	TTC _theoretical_ (μM GSH)	TTC _experimentally_ (μM GSH)
40 μL 1 mM GSH 40 μL 1 mM CYS	10.0 14.8	24.8	25.7 ± 0.1
40 μL 1 mM GSH 40 μL 1 mM NAC	10.0 10.2	20.2	21.1 ± 0.2
40 μL 1 mM CYS 40 μL 1 mM NAC	14.8 10.2	24.8	25.2 ± 0.1
40 μL 1 mM GSH 40 μL 1 mM NAC 40 μL 1 mM CYS	10.0 10.2 14.8	35.0	36.1 ± 0.3

p = 0.05, F_exp_ = 0.0042, F_crit(Table)_ = 10.13, F_exp_ < F_crit(Table)_.

Figure S6 The molecular electrostatic potential (MEP) of (3) derived through density functional theory (DFT) calculations employing the wb97xd functional and the 6–311++G(d,p) basis set.

Figure S7 The frontier molecular orbital compositions for (3), as determined using DFT/wb97xd/6-311++G(d,p) level of theory.

Figure S8 Comparison of the crystal structure of glutation in complex with the Glutathione S-transferases (GSTs) from Fasciola hepatica (olive sticks) [25] (2FHE) with the re-docked outcomes of compound (3) into the unliganded 2FHE target (blue sticks).

## Figures and Tables

**Table 1:** Analytical parameters for the fluorometric determination of biothiols by compound 3 and the GETC coefficients of the biothiols.

Biothiols	Linear Range (μM)	Slope	Intercept	*r*	RSD (%) Inter-/Intra-days	LOD (μM)	GETC
GSH	10–70	1.30	0.36	0.9990	0.95/1.21	0.11 (0.32;1.42)*	1.00
NAC	10–60	1.12	0.86	0.9991	1.22/1.63	0.34 (0.26;1.66)*	0.86
CYS	10–70	1.65	0.31	0.9991	0.81/1.29	0.08 (0.31; 0.80)*	1.27

* In parentheses, LOD values according to DTNB-Au-NP and the original Elmann methods, respectively.

LOD = 3*s*_bl_*/m* (s_bl_: the standard deviation of blank and m: is the slope value of the calibration curve).

**Table 2:** Comparison of compound 3 and other similar fluorogenic biothiol probes.

Fluorogenic probes	Response time (min)	Biothiols	LOD (μM)	Reference
Cyanine-based (1)	3	GSH	---	[33]
Cyanine-based (2)	2	GSH	5	[34]
Hemicyanine-based (3)	1	CYS	---	[35]
Xanthene-based (4)	<1	GSH	0.01	[36]
Pyranocoaumarin-based (5)	<1	CYS GSH	0.08 0.11	[37]
Other (6)	<1	GSH	0.148	[38]
Compoud (3)	≤1	GSH CYS	0.11 0.08	This work

**Table 3:** Measurement of quantities of GSH mixed with possible interfering compounds (N = 3).

Composition of mixture	GSH added (μM)	Found (μM)	Relative error (%)
0.2 mL 1 mM GSH 0.04 mL 1 mM UA	50.0	49.21	−1.63
0.2 mL 1 mM GSH 0.04 mL 1 mM CT	50.0	48.73	−2.61
0.2 mL 1 mM GSH 0.04 mL 1 mM BSA	50.0	49.13	−1.82
0.2 mL 1 mM GSH 0.04 mL 1 mM AA	50.0	48.32	−3.41
0.2 mL 1 mM GSH 0.04 mL 1 mM Glycine	50.0	47.54	−4.61

**Table 4:** Results obtained with the standard addition of the studied biothiols to fetal bovine serum (FBS) (N = 3).

GSH addition to FBS	added concn. (μM)	10.00
	mean (μM)	10.51
	SD^a^	0.35
	% RSD	3.33
	% REC	105.1
NAC addition to FBS	added concn. (μM)	10.00
	mean (μM)	9.39
	SD^a^	0.24
	% RSD	2.56
	% REC	93.9
CYS addition to FBS	added concn. (μM)	10.00
	mean (μM)	9.66
	SD^a^	0.41
	% RSD	4.24
	% REC	96.6

a Standard deviation.
