# Supplementary figures and images for: A fast and responsive turn-on fluorescent probe based on a quinone conjugated alkoxy derivative for biothiols and a cellular imaging study
Source: Turk J Chem. 2024 Nov 11;48(6):830–42. doi: 10.55730/1300-0527.3702 (PMC11706294; doi:10.55730/1300-0527.3702)

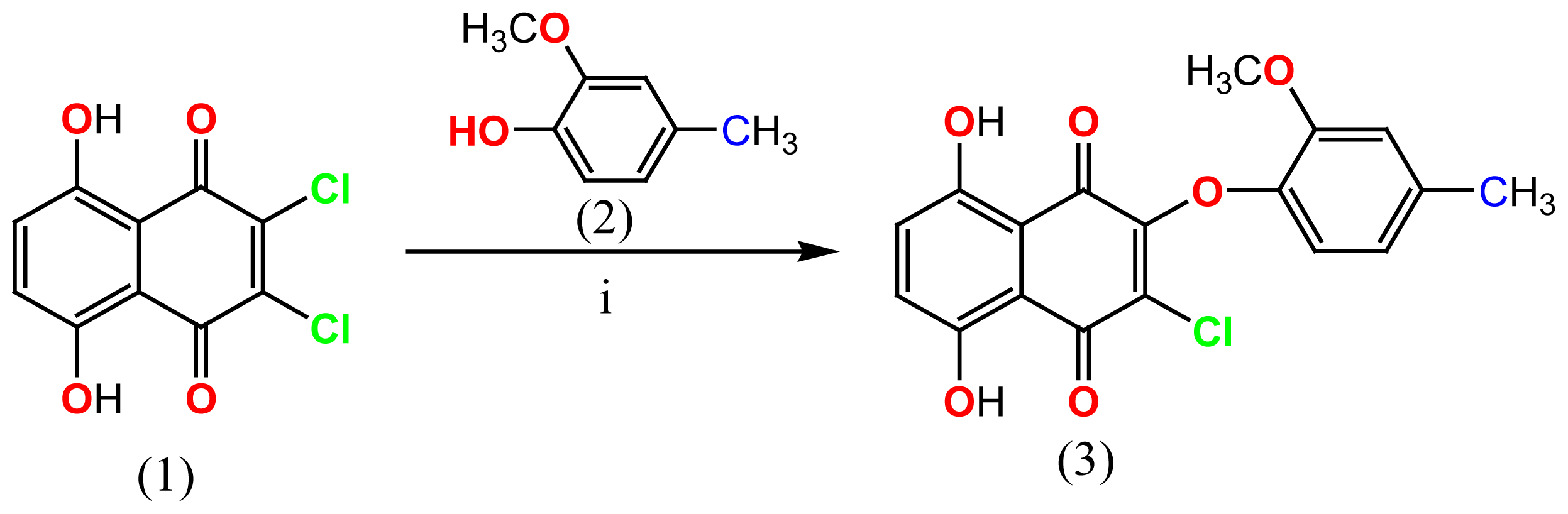

Supplement: Scheme S — Preparation of quinone based probe. i: DMSO/Na2CO3. [file tjc-48-06-830s1.tif]

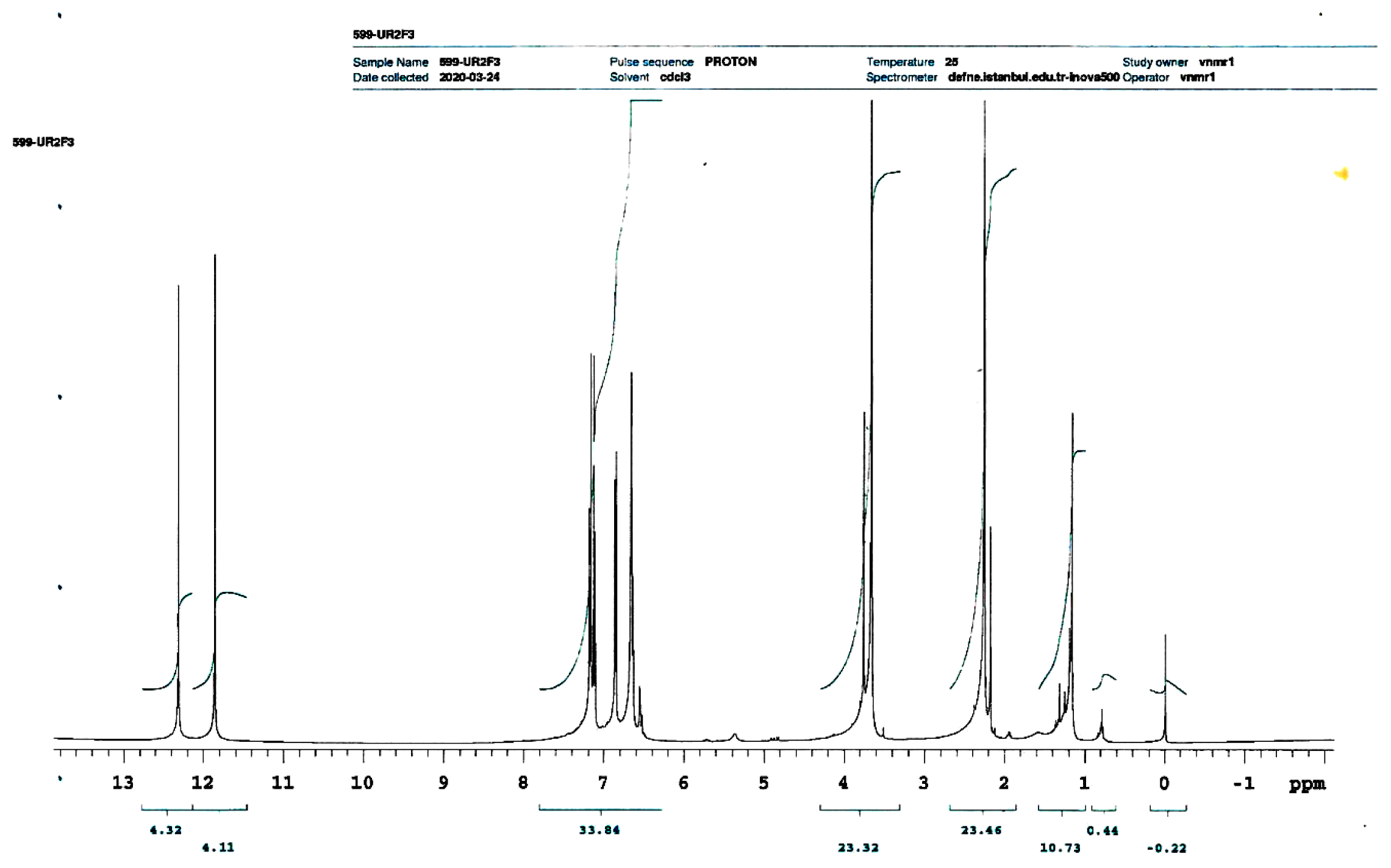

Supplement: Figure S1 — 1H-NMR spectrum of compound (3). [file tjc-48-06-830s2.tif]

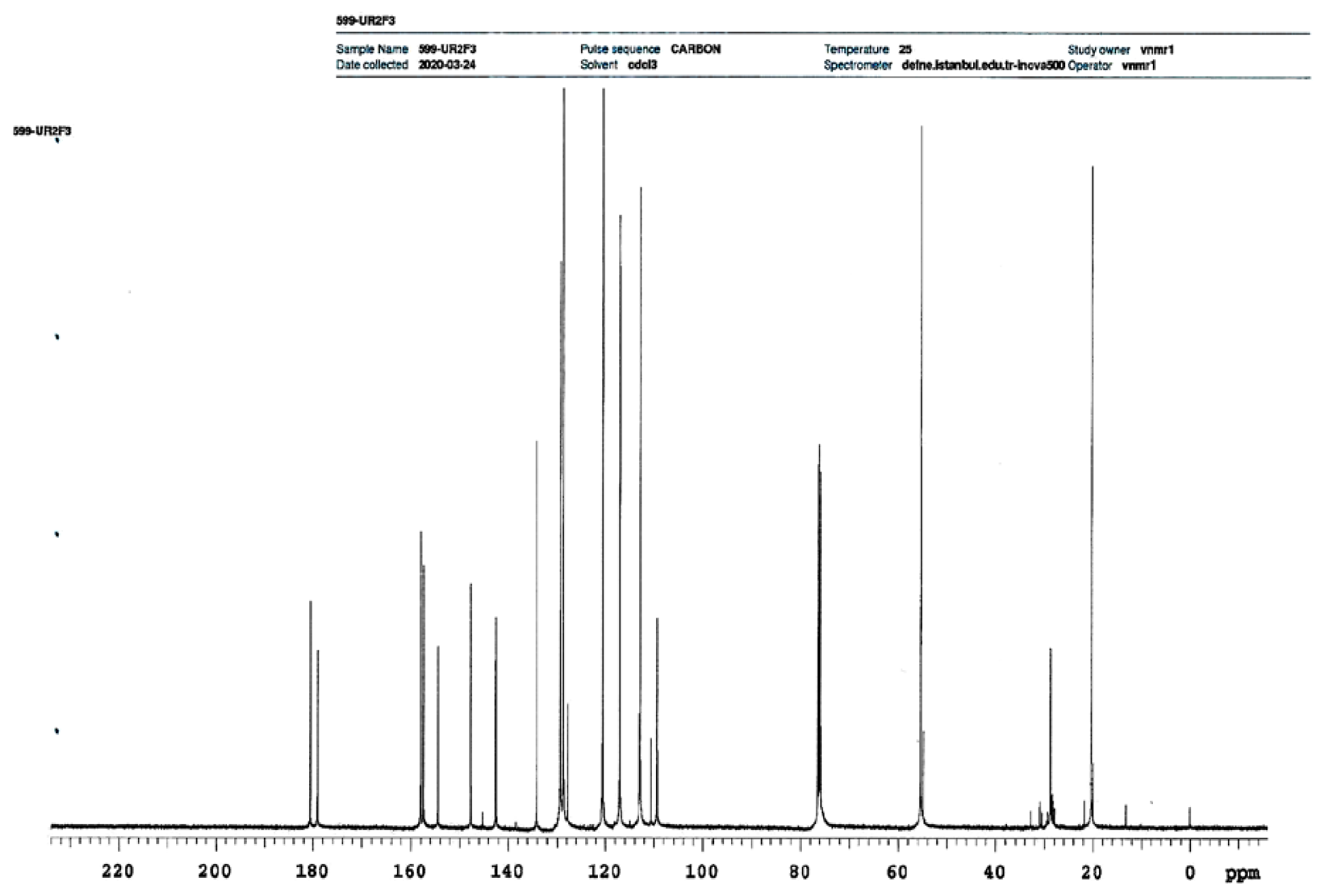

Supplement: Figure S2 — 13C-NMR spectrum of compound (3). [file tjc-48-06-830s3.tif]

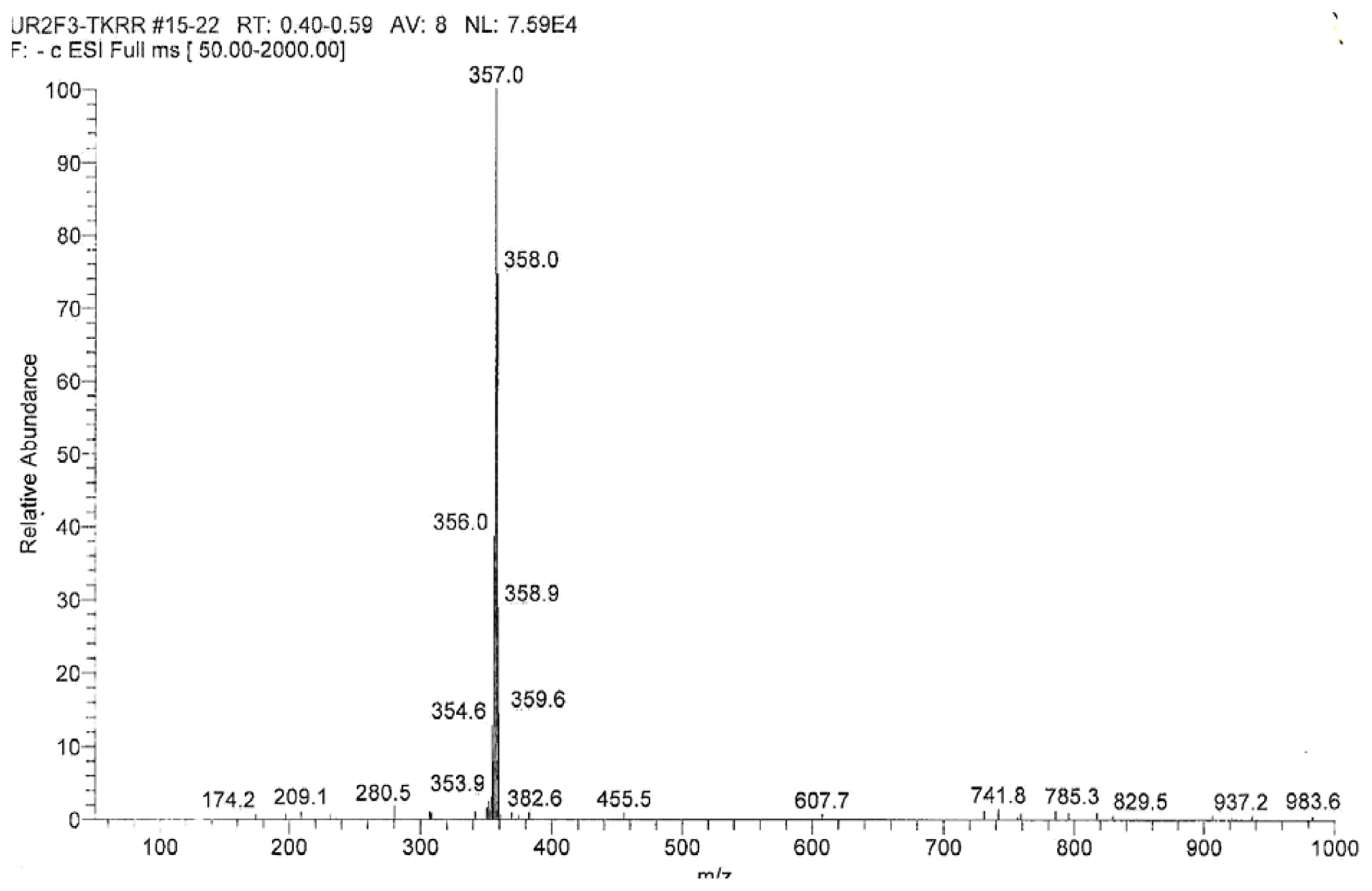

Supplement: Figure S3 — Mass spectrum of compound (3). [file tjc-48-06-830s4.tif]

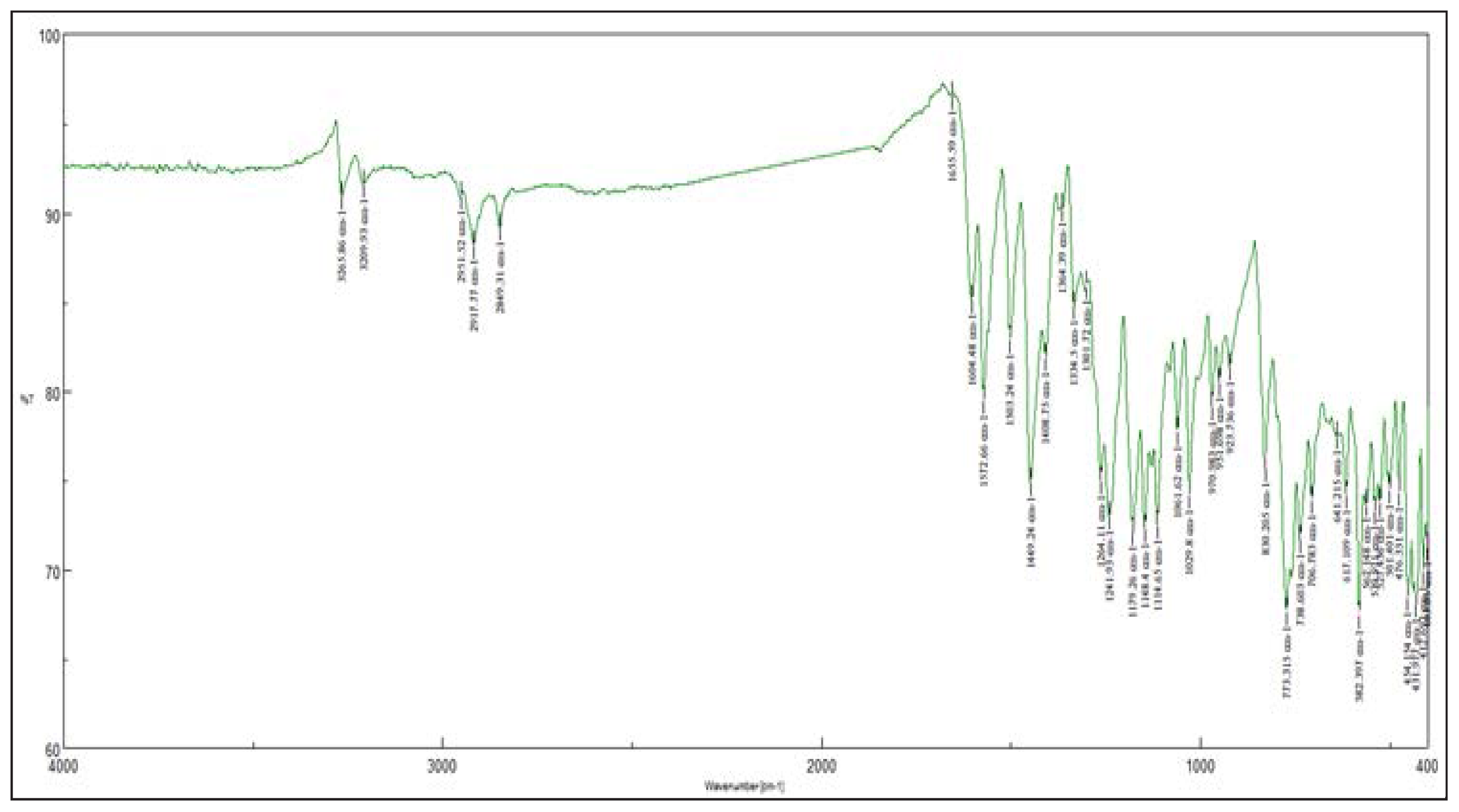

Supplement: Figure S4 — FT-IR spectrum of compound (3). [file tjc-48-06-830s5.tif]

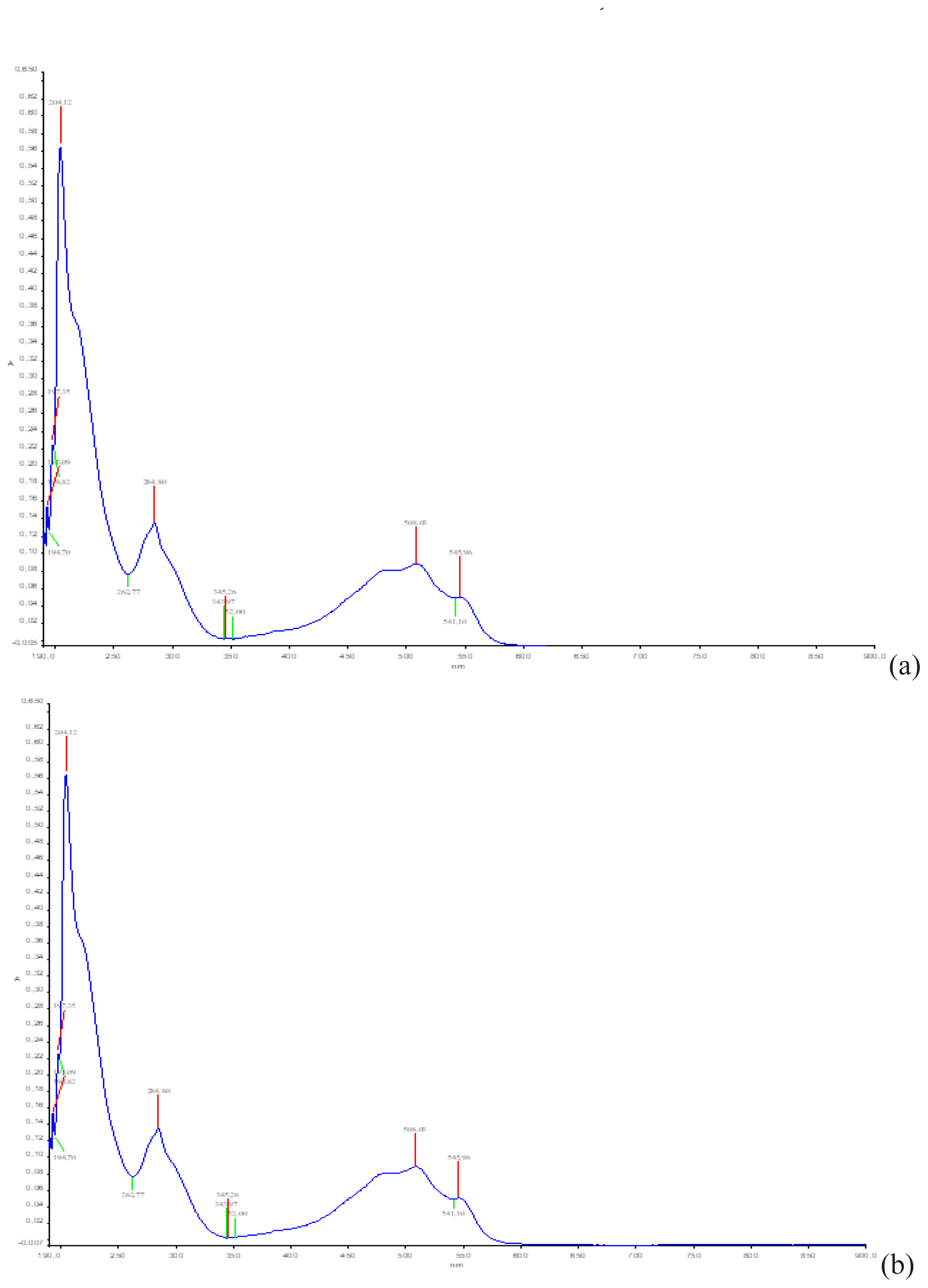

Supplement: Figure S5 — Absorption spectra of compound (3) in chloroform (a) and ethanol (b). [file tjc-48-06-830s6.tif]

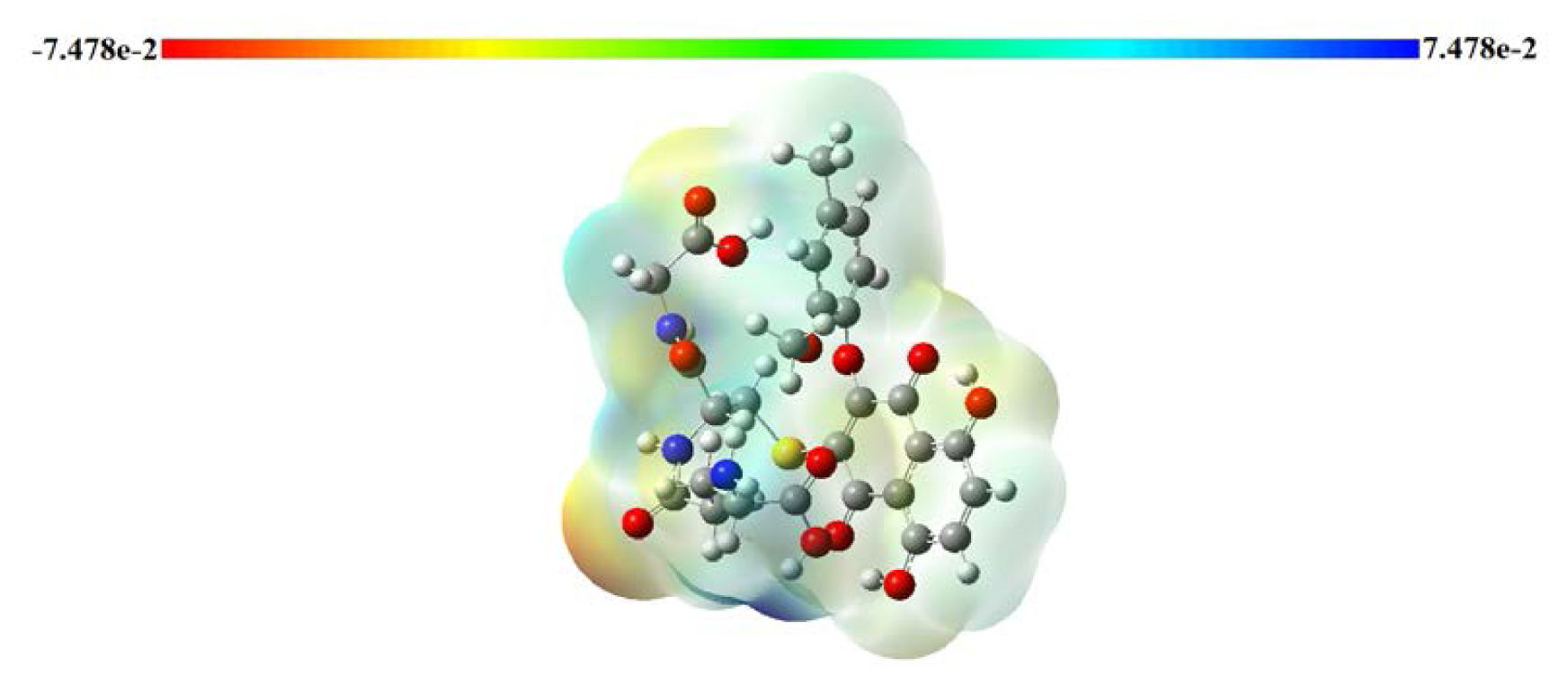

Supplement: Figure S6 — The molecular electrostatic potential (MEP) of (3) derived through density functional theory (DFT) calculations employing the wb97xd functional and the 6–311++G(d,p) basis set. [file tjc-48-06-830s7.tif]

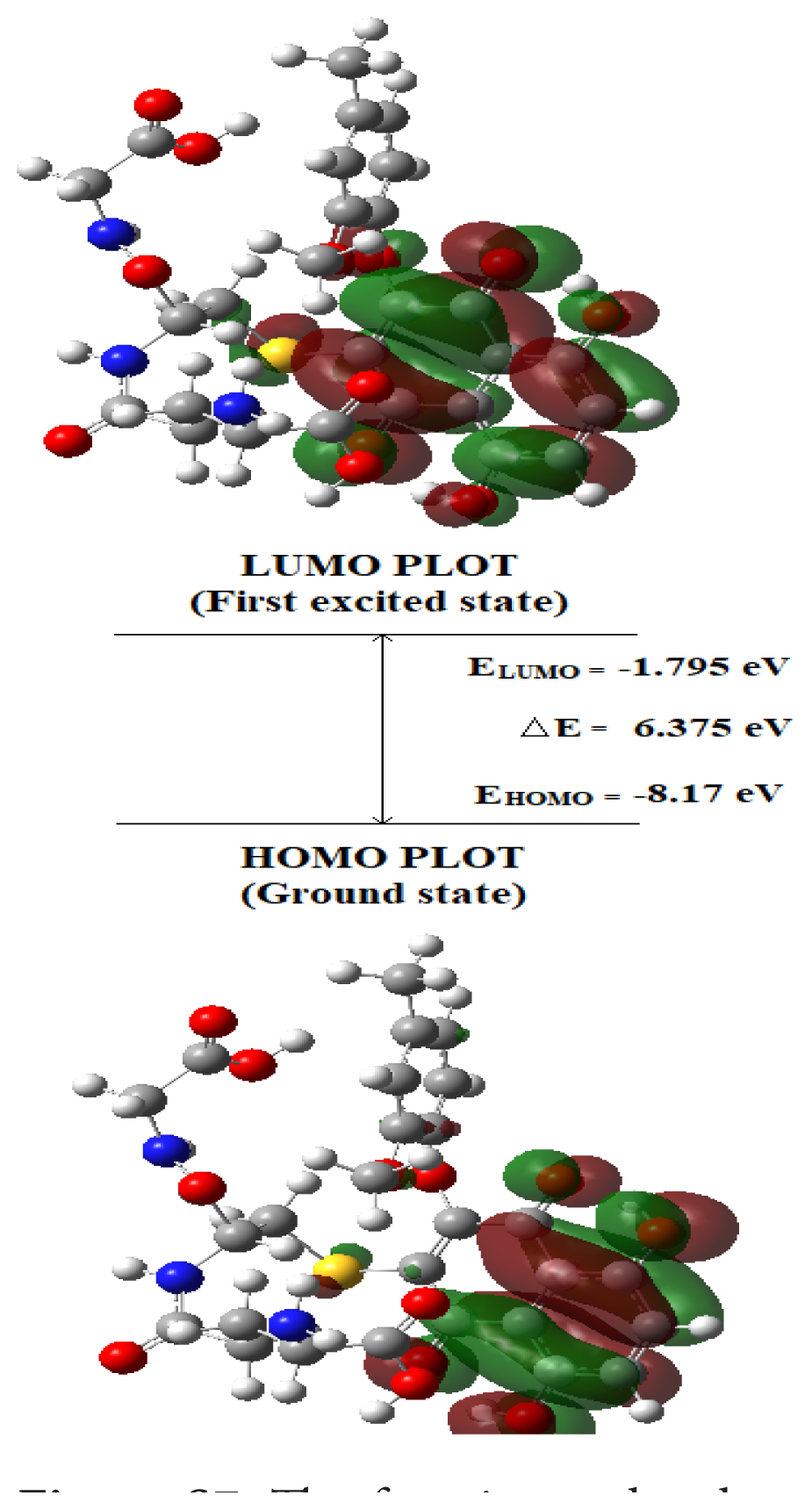

Supplement: Figure S7 — The frontier molecular orbital compositions for (3), as determined using DFT/wb97xd/6-311++G(d,p) level of theory. [file tjc-48-06-830s8.tif]

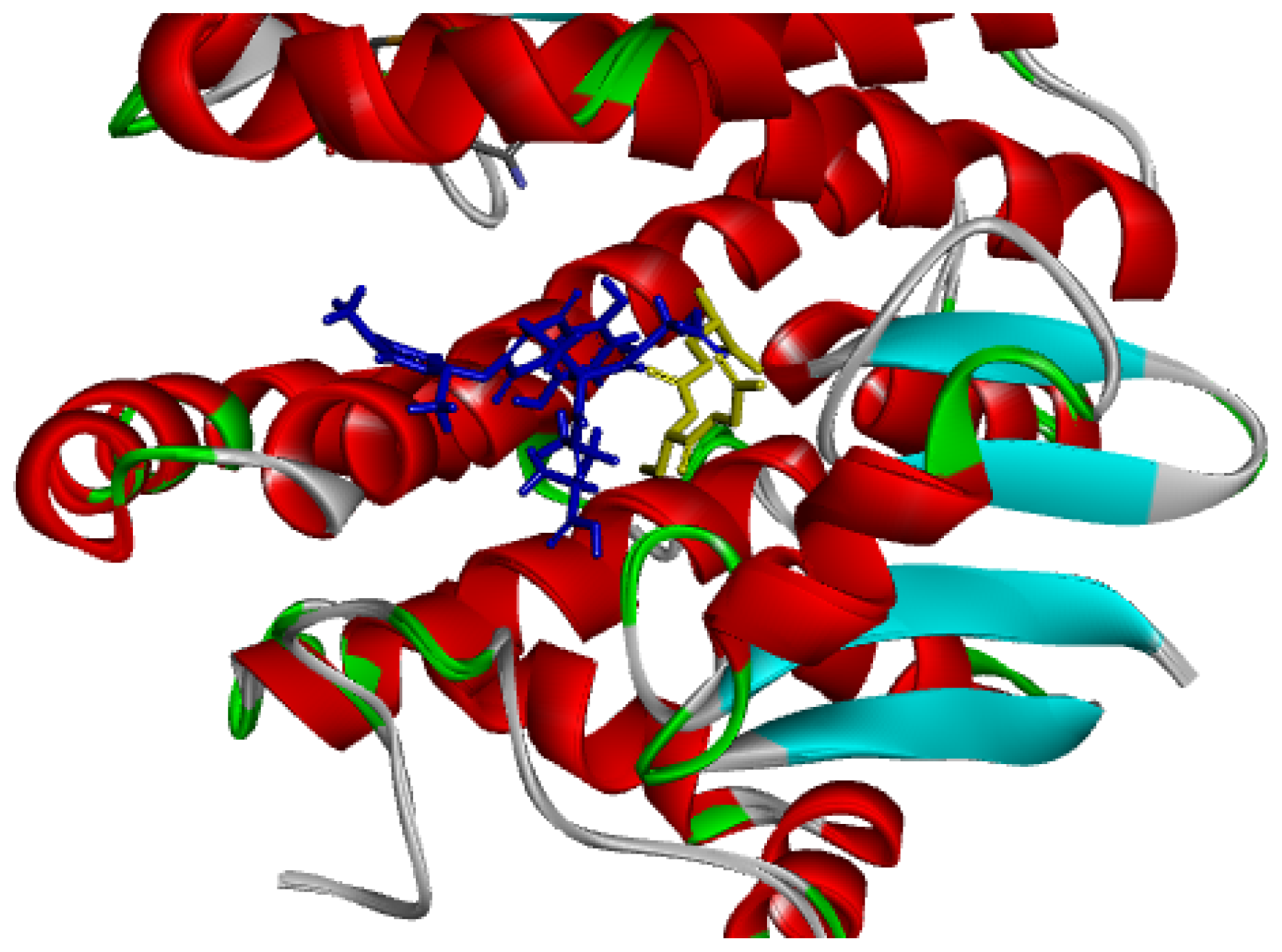

Supplement: Figure S8 — Comparison of the crystal structure of glutation in complex with the Glutathione S-transferases (GSTs) from Fasciola hepatica (olive sticks) [25] (2FHE) with the re-docked outcomes of compound (3) into the unliganded 2FHE target (blue sticks). [file tjc-48-06-830s9.tif]
